# Early and Phasic Cortical Metabolic Changes in Vestibular Neuritis Onset

**DOI:** 10.1371/journal.pone.0057596

**Published:** 2013-03-07

**Authors:** Marco Alessandrini, Marco Pagani, Bianca Napolitano, Alessandro Micarelli, Matteo Candidi, Ernesto Bruno, Agostino Chiaravalloti, Barbara Di Pietro, Orazio Schillaci

**Affiliations:** 1 Department of Medical Science and Translational Medicine, “Tor Vergata” University, Rome, Italy; 2 Institute of Cognitive Sciences and Technologies, CNR, Rome, Italy; 3 Department of Psychology, “Sapienza” University and IRCCS Santa Lucia, Rome, Italy; 4 Department of Biopathology and Diagnostic Imaging, “Tor Vergata” University, Rome, Italy; 5 IRCCS Neuromed, Pozzilli, Italy; University of Manchester, United Kingdom

## Abstract

Functional brain activation studies described the presence of separate cortical areas responsible for central processing of peripheral vestibular information and reported their activation and interactions with other sensory modalities and the changes of this network associated to strategic peripheral or central vestibular lesions. It is already known that cortical changes induced by acute unilateral vestibular failure (UVF) are various and undergo variations over time, revealing different cortical involved areas at the onset and recovery from symptoms. The present study aimed at reporting the earliest change in cortical metabolic activity during a paradigmatic form of UVF such as vestibular neuritis (VN), that is, a purely peripheral lesion of the vestibular system, that offers the opportunity to study the cortical response to altered vestibular processing. This research reports [^18^F]fluorodeoxyglucose positron emission tomography brain scan data concerning the early cortical metabolic activity associated to symptoms onset in a group of eight patients suffering from VN. VN patients’ cortical metabolic activity during the first two days from symptoms onset was compared to that recorded one month later and to a control healthy group. Beside the known cortical response in the sensorimotor network associated to vestibular deafferentation, we show for the first time the involvement of Entorhinal (BAs 28, 34) and Temporal (BA 38) cortices in early phases of symptomatology onset. We interpret these findings as the cortical counterparts of the attempt to reorient oneself in space counteracting the vertigo symptom (Bas 28, 34) and of the emotional response to the new pathologic condition (BA 38) respectively. These interpretations were further supported by changes in patients’ subjective ratings in balance, anxiety, and depersonalization/derealization scores when tested at illness onset and one month later. The present findings contribute in expanding knowledge about early, fast-changing, and complex cortical responses to pathological vestibular unbalanced processing.

## Introduction

For many years, research has focused on labyrinth, brainstem and cerebellum structures to elucidate the neural correlates of vestibular functions. However, since the vestibular system is highly convergent and multimodal from the second-order sensory neuron, spatial orientation and perception of movement require processing of vestibular information also at the cortical level in association with visual, somato-sensory and motor systems and comparatively little is known about cortical representation of the vestibular system [1].

Animal experiments have shown that there is no primary vestibular cortex that receives projections exclusively from vestibular afferents [2], [3]. Instead, several multimodal sensory areas have been identified that integrate vestibular, visual and somato-sensory signals. In humans, these areas have been partly confirmed by intraoperative cortical stimulation, clinical studies in patients with acute cortical lesions and functional imaging including blood flow measurements with single photon emission computed tomography (SPECT) [4]–[6], functional magnetic resonance imaging (fMRI) [7], [8] and positron emission tomography (PET) [9], [10]. These latter studies aimed to identify a network of cortical areas activated during vestibular stimulation via either caloric or galvanic stimuli.

Subsequent investigations analysed the effect of a peripheral vestibular lesion on cerebral cortex processing of vestibular stimuli and reported metabolic changes in vestibular cortex during acute vestibular neuritis (VN) using [^18^F]fluorodeoxyglucose positron emission tomography (FDG-PET) [11]–[13] as well as structural changes at the cortical and cerebellar level using voxel-based morphometry [14].

Although a recent review of the structural and functional literature together with evidence from neurologic patients suggested that is at present difficult to conclude whether there is a human homologue of monkeys’ Parieto-Insular-Vestibular-Cortex (PIVC) and whether this may fall into posterior insula, inferior parietal lobule, superior temporal gyrus or at the junction between these regions [15], a recent meta-analysis on available functional literature using activation likelihood estimation to locate cortical neural regions associated to vestibular processing suggested that right hemispheric parietal opercular area may be considered as the human homologue of non-human primates PIVC [16].

VN, a disease that we have already studied and described in previous papers [17], [18], is a purely peripheral lesion of the vestibular system. It constitutes an ideal “experimental model” of a partial vestibular de-afferentation, useful for observing the effect of vestibular de-afferentation on the cerebral cortex during the acute phase. VN is defined as a sudden, usually partial, unilateral failure of the peripheral vestibular organ without hearing impairment and no signs of brainstem dysfunction. It is the second most common cause of vertigo, it tends to occur most frequently in 40–50 years old, affects both sexes and all ethnic groups and is often associated with recent or concurrent upper respiratory infections but its pathogenesis is unknown [19]. Characteristic signs and symptoms of VN include a sudden onset of severe rotational vertigo associated with spontaneous nystagmus, nausea, vomiting, emotional disturbances, postural instability and no other neurologic or cochlear symptoms and findings [19]–[21]. As known, this symptomatology is very severe in the first few days due to a sudden loss of environment landmarks with a great emotional impact. It has been shown that 6 days after VN symptoms onset the regional cerebral glucose metabolism (rCGM) is significantly increased in multisensory vestibular cortical and subcortical areas (parietoinsular vestibular cortex in the posterior insula, posterolateral thalamus, anterior cingulate gyrus [Brodmann area 32/24], pontomesencephalic brainstem, hippocampus) and that there is a simultaneous significant rCGM decrease in visual (Brodmann area 17 to 19) and somatosensory cortices in the postcentral gyrus as well as in parts of the auditory cortex (transverse temporal gyrus) [11]. Furthermore, a voxel-based structural morphometry study confirmed that the response to VN is reflected in a change in the vestibular system that shows a white matter increase in the commissural fibers (probably as a direct consequence of an increased internuclei vestibular crosstalk of the medial vestibular nuclei), in the somatosensory system due to an elevated processing of proprioceptive information in the right gracile nucleus (possibly to regain postural stability), and a selective bilateral structural increase in the area of MT/V5 in VN patients with a residual peripheral vestibular hypofunction (a possible result of an increased importance of visual motion processing) [22].

By using FDG-PET the present study aimed at detecting even faster and phasic cortical metabolic changes during the first two days from the onset of symptoms in VN patients. Measuring the earliest metabolic change associated to altered vestibular inflow seems particularly important as it is known that the metabolism of the cortex may rapidly adapt to the new uncontrolled peripheral inflow, leaving no clear sign of altered neural response. We compared this early pattern of metabolic activity with patients’ cortical activity one month after symptoms onset and with a control group. The arbitrary choice to test patients one month after symptoms onset was based on the idea to provide a description of earlier metabolic effects than those already available in literature.

Furthermore, in order to be able to get a clinical, cognitive and functional description of patients’ state, a number of subjective measures concerning 1) balance symptoms [23], 2) anxiety symphtoms [24] and 3) depersonalization/derealization dimensions in clinically anxiety states [25], were collected from the patients.

## Subjects and Methods

### Ethics Statement

The Ethics Committee of the Tor Vergata University School of Medicine approved the protocol research “Cerebral metabolism changes, in patients affected by acute vestibular failure compared with normal subjects, using [^18^F]fluorodeoxyglucose positron emission tomography”. The study adhered to the principles of the Declaration of Helsinki and all of the participants provided written informed consent after receiving a detailed explanation of the study.

### Diagnosis

Thorough neurological and neurootological examination was performed and VN was diagnosed according to the generally accepted criteria: (1) sudden onset of vertigo and neurovegetative symptoms; (2) static and dynamic ataxia; (3) spontaneous, one-way and persistent nystagmus with slow phase toward the affected ear detected by means of binocular electrooculography analysis; absence of (4) cochlear and (5) associated neurological symptoms or signs [19], [26]. T2-weighted and/or diffusion-weighted magnetic resonance imaging (MRI) sequences of the brainstem were acquired with a 1.5 T clinical MRI scanner to exclude the possibility that pseudoneuritis affected the vestibular nucleus or vestibular afferents within the brainstem.

### Patients and PET/CT Examination

Eight right handed patients (five females; three males; mean age 48±7 years) presenting with the first and sole episode of VN, right-sided, underwent a FDG-PET brain scan using 3D-mode standard technique during the acute phase of VN (48 hours ±6 hours). After the first brain PET/CT scan (PET0), all the patients underwent a second PET/CT scan 1 month (±2 days) later (PET1). Thirty healthy volunteers (sixteen women and fourteen men, mean age 49.5±12 years) undergoing FDG PET and found to be completely negative for various diseases served as controls (CG) [27]. Exclusion criteria were presence of major systemic illness, major vision disturbances, psychiatric illnesses, epilepsy, head trauma, Parkinsonism, previous stroke or TIA, presence of brain masses as well as the current use of benzodiazepines and tricyclic antidepressants.

### PET Scanning

The PET/CT system Discovery ST16 (GE Medical Systems, TN, USA) was used for the whole population in exam. This system combines a high-speed ultra 16-detector-row (912 detectors per row) CT unit and a PET scanner with 10080 bismuth germanate (BGO) crystals in 24 rings with a 128×128 matrix. Crystal size 6, 2×6, 2×30 mm, coincidence window 11,7 nsec, system sensitivity 9,3 cps/kBq in 3D mode, dispersion fraction 44%, maximum count rate in cps at 50% dead time 63 kcps @ 12 kBq/mL (3-D), axial FWHM 1 cm radius 5,2 mm in 3D mode, axial FOV 157 mm.

All patients fasted for at least 5 h before F18-FDG i.v. injection; serum glucose level was normal in all of them. Patients were injected with 3 MBq/Kg (210–350 MBq) of ^18^F-FDG i.v. and hydrated (500 ml of i.v. saline sodium chloride (NaCl) 0.9%) to reduce pooling of the radiotracer in the kidneys while laying down in a noiseless and semi-darkened room with their eyes open and without any artificial stimulation.

Patients and controls with diabetes, neurological and psychiatric disorders, history of oncologic disease, HIV, epilepsy and surgery, radiation or trauma to the brain were excluded from the study. All subjects were negative for cochlear, vestibular, or central nervous system disorders. Except for the antiemetic drug ondansetron, given intravenously at a dosage of 8 mg/die for the first three days from the onset of acute symptoms, patients were not taking any medications. Moreover, we excluded from our study all the patients in treatment with drugs that could interfere with ^18^F-FDG uptake and distribution in the brain [28]. No patients were pregnant or breastfeeding.

### Statistical Analysis

Differences in brain ^18^F-FDG uptake were analyzed using statistical parametric mapping (SPM2, Wellcome Department of Cognitive Neurology, London, UK) implemented in Matlab 6.5 (Mathworks, Natick, Massachusset, USA). PET data were subjected to affine and non-linear spatial normalization into the MNI space. The spatially normalized set of images were then smoothed with a 12 mm isotropic Gaussian filter to blur individual variations in gyral anatomy and to increase the signal-to-noise ratio. Images were globally normalized using proportional scaling to remove confounding effects to global cerebral glucose metabolism changes, with a threshold masking of 0.8. The resulting statistical parametric maps, SPM{t}, were transformed into normal distribution (SPM{z}) unit. Correction of SPM coordinates to match the Talairach coordinates was achieved by the subroutine implemented by Matthew Brett (http://www.mrc-cbu.cam.ac.uk/Imaging). Brodmann areas (BAs) were then identified at a range of 0 to 3 mm from the corrected Talairach coordinates of the SPM output isocentres, after importing them by Talairach client (http://www.talairach.org/index.html). Thresholds equal or lower than p<0.001 corrected at cluster level were accepted as significant. Only those clusters containing more than 125 (5×5×5 voxels, i.e. 11×11×11 mm) contiguous voxels were accepted as significant, based on the calculation of the partial volume effect resulting from the spatial resolution of the PET camera (about the double of FWHM).

The following voxel-based comparisons were assessed: (1) PET0 *vs* PET1 and viceversa; (2) CG *vs* PET0 and *viceversa* (3) CG vs PET1 and *viceversa*. The PET0 *vs* PET1 comparison was performed using a ‘two conditions: one scan/condition, paired t-test’ design model. The CG *vs* PET0/PET1 comparisons were performed by means of the ‘compare populations: 1 scan/subject (Ancova)’ option. In this latter comparison age and sex were used as covariates in the SPM analyses.

### Validated Questionnaires

Patients’ subjective responses to the following questionnaires were collected at PET0 and PET1:

1. A standardized questionnaire of balance symptoms that includes nine items to report each of the balance symptoms, with no/yes answers. A “no” response is scored 0 points and a “yes” response is scored 1 point except for vertigo, which is scored 2 points; frequent falls are considered when occurring at least once per month and frequent stumbles when occurring at least once per week. The total score is calculated by adding-up all the points (range 0–10). The authors suggested that a score higher than 3 points could be related to balance disorders [23];
2. The Zung Instrument for Anxiety Disorders, a 20- item scale with some of the items keyed positively and some negatively on a four-point scale ranging from 1 =  none or a little of the time to 4 =  most or all of the time. The final score range from 20–80, a score between 20 and 44 is considered in the normality range, 45–59 is mild to moderate anxiety, 60–74 is severe, and 75–80 is very severe [24];
3. The 28-item depersonalization/derealization inventory (DD) by Cox and Swinson (2002), which is a tool designed to assess symptoms of depersonalization/derealization in clinically anxiety states, more than in a dissociative disorders context. Severity of each item is coded on a scale where 0 =  does not occur, 1 =  mild, 2 =  moderate, 3 =  severe and 4 =  very severe. The total score is calculated by adding all the points (range 0–112). The higher scores are related to a higher frequency and/or severity of DD symptoms. No cutoff score has been suggested [25].

To evaluate the correlations between brain metabolism and subjective variables, the clusters of voxels found to be significantly different at PET0– PET1 and PET1– PET0 comparisons (see Table 1) were segmented and the semi-quantitative raw data values in each of them were individually normalized to the relative FDG uptake in the cerebellum. All values were then transformed into z-scores and submitted to statistical analyses seeking for correlations with DD, Balance and Anxiety – total scores. The correlation analyses have been performed considering the values at PET0 and PET1 (as well as the relative tests scores) as a continuum.

**Table 1:** Numerical results of SPM comparisons between ^18^F-FDG uptake in PET0 (n = 8) and PET1 (n = 8).

Comparison	Cluster level			Voxel level			
	Clusterextent	Correctedp value	Corticalregion	Z score ofmaximum	Talairachcoordinates	Cortical region	BA
**PET0– PET1**	727	0.000	R	3.26	18,1,−19	Uncus	34
			R	3.19	24,6, −32	Superior Temporal Gyrus	38
			R	2.97	30,18, −24	Inferior Frontal Gyrus	47
			R	2.96	20, −13, −20	Parahippocampal Gyrus	28
	364	0.000	R	2.85	10,7, −17	Subcallosal Gyrus	25
			R	2.64	10,13, −19	Rectal Gyrus	11
**PET1– PET0**	8143	0.000	L	3.27	−4, −64,49	Precuneus	7
			L	3.17	−46, −46,48	Inferior Parietal Lobule	40
			R	3.11	2, −90,17	Cuneus	18
			L	3.10	−2, −45,63	Paracentral Lobule	5
			R	3.03	4, −42,57	Paracentral Lobule	5
			L	3.03	−2, −97,3	Cuneus	18
			R	3.03	22, −50,47	Precuneus	7
			R	3.02	8, −92,27	Cuneus	19
			L	2.97	0, −97,0	Cuneus	17

A value of p≤0.001, corrected for multiple comparison at cluster level, was accepted as statistically significant. In the ‘cluster level’ section on left, the number of voxels, the corrected p value of significance and the cortical region where the voxel is found, are all reported for each significant cluster. In the ‘voxel level’ section, all of the coordinates of the correlation sites (with the Z-score of the maximum correlation point), the corresponding cortical region and BA are reported for each significant cluster. L, left; R, right; BA, Brodmann’s area. In the case that the maximum correlation is achieved outside the grey matter, the nearest grey matter (within a range of 3×mm) is indicated with the corresponding BA.

## Results

### PET

**Figure 1:**
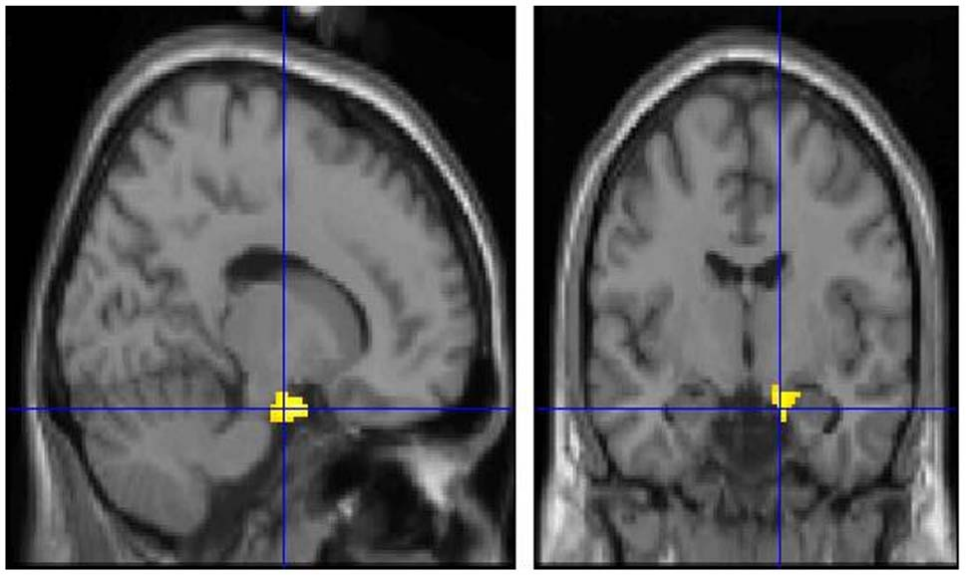
T1 MRI superimposition showing the cluster of voxels in the right parahippocampal gyrus (BAs 34 and 28) in which 18F-FDG uptake was significantly higher at PET0 (n = 8) as compared to PET1 (n = 8) (on the left sagittal and on the right coronal projections). Coordinates and regional details are presented in Table 1.

**Figure 2:**
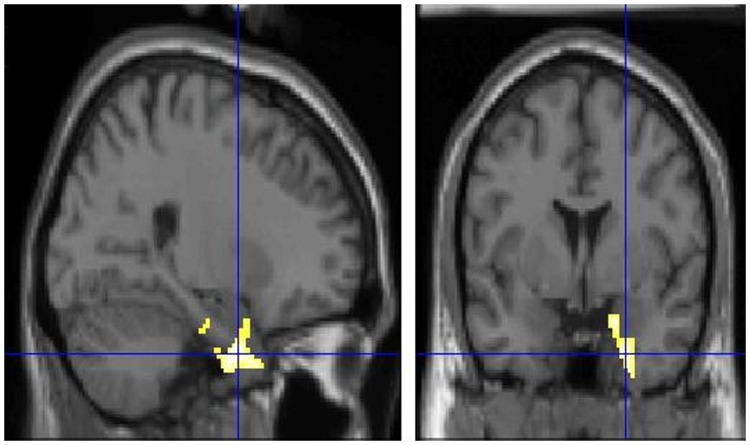
T1 MRI superimposition showing the cluster of voxels in the superior temporal gyrus (BA 38) in which 18F-FDG uptake was significantly higher at PET0 (n = 8) as compared to PET1 (n = 8) (on the left sagittal and on the right coronal projections). Coordinates and regional details are presented in Table 1.

A significantly higher glucose metabolism in right parahippocampal (BAs 34, 28) (Figure 1), right orbitofrontal (BAs 47, 11), right subcallosal (BA 25) cortex and right superior temporal gyrus (BA 38) (Figure 2) was found at PET0 as compared to PET1 (Table 1).

As compared to PET0, patients at PET1 showed a significantly increased glucose metabolism in bilateral somatosensory associative (BA 5) in bilateral parietal (BA7, 40) and bilateral visual (BAs 17, 18, 19) cortices (Table 1).

In the comparison with healthy volunteers PET0 patients showed a significantly reduced glucose metabolism in bilateral visual (BAs 17, 18, 19), bilateral parietal (BA 7) and in left primary somatosensory (BA 2) cortices. In the opposite comparison a significantly increased glucose metabolism in right superior temporal gyrus (BA 38) and in left posterior insular cortex (BA 13) was found (Table 2).

**Table 2:** Numerical results of SPM comparisons between ^18^F-FDG uptake in CG (n = 30) and PET0 (n = 8).

Comparison	Cluster level			Voxel level			
	Clusterextent	Correctedp value	Corticalregion	Z score ofmaximum	Talairachcoordinates	Cortical region	BA
**CG - PET0**	22280	0.000	L	5.22	−22, −92, −16	Fusiform Gyrus	18
			L	4.68	−22, −86,34	Cuneus	19
			R	4.60	22, −94, −14	Fusiform Gyrus	18
			R	4.60	16, −95, −4	Cuneus	17
			R	4.58	28, −73,50	Precuneus	7
			R	4.49	12, −86,37	Cuneus	19
			L	4.20	−2, −65,53	Precuneus	7
			L	3.89	−51, −27,51	Postcentral Gyrus	2
**PET0 - CG**	1032	0.03	R	3.88	38,7, −21	Superior Temporal Gyrus	38
			L	3.29	−40,1, −12	Posterior Insula	13

A value of p≤0.05, corrected for multiple comparison at cluster level, was accepted as statistically significant. In the ‘cluster level’ section on left, the number of voxels, the corrected p value of significance and the cortical region where the voxel is found, are all reported for each significant cluster. In the ‘voxel level’ section, all of the coordinates of the correlation sites (with the Z-score of the maximum correlation point), the corresponding cortical region and BA are reported for each significant cluster. L, left; R, right; BA, Brodmann’s area. In the case that the maximum correlation is achieved outside the grey matter, the nearest grey matter (within a range of 3 mm) is indicated with the corresponding BA.

No statistically significant differences were found when patients at PET1 were compared to healthy volunteers.

### Questionnaires

**Figure 3:**
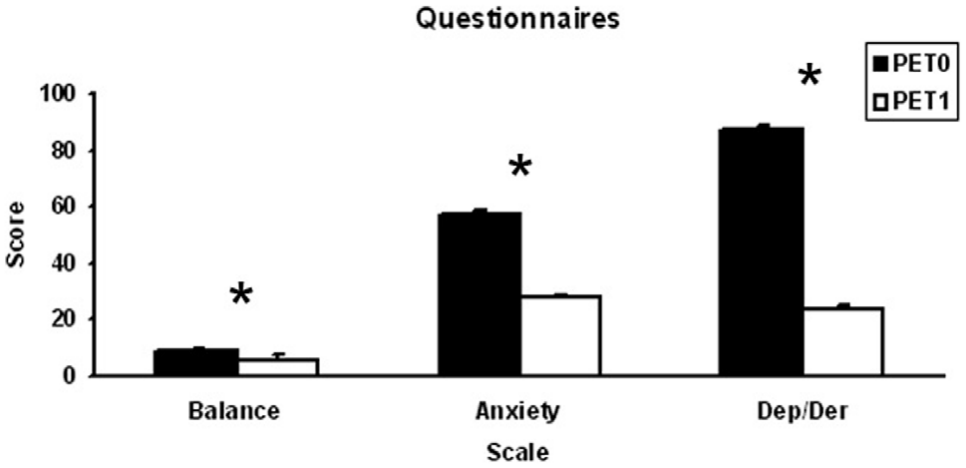
Mean and standard deviations of patients scores in the questionnaires on Balance (Gomez-Alvarez and Jauregui-Renaud, 2011), Anxiety (Zung, 1971), and Depersonalization/Derealization scales (Dep/Der, Cox and Swinson, 2002) at PET0 and PET1.

Direct t-test comparisons between the subjective scores at PET0 and PET1 showed a decrease of Balance disorders (t(7) = 4.2488, p<0.01), Anxiety (t(7) = 44.0870, p<0.01) and Depersonalization/Derealization (t(7) = 151.5142, p<0.01) scores (Figure 3).

The correlation analyses resulted in significant (p<0.05) negative correlations between the metabolism in the cluster (comprising mainly visual and parietal regions) resulting from the difference between PET1 and PET0 (hypometabolism during the acute phase of VN) and the scores of DD (r = −0.63), Balance (r = −0.55) and Anxiety (r = −0.62). Significant positive correlations were found only between the metabolism in the clusters (comprising parahippocampal, temporal and anterior cingulate regions) resulting from the differences between PET0 and PET1 (hypermetabolism in the acute phase of VN) and DD (r = 0.51) while the correlation with Zung and Gomez scores resulted not significant (r = 0.44, r = 0.17 respectively).

**Figure 4:**
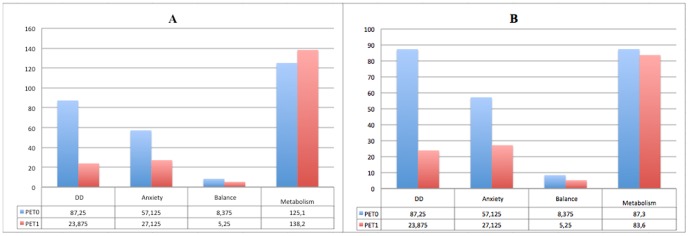
Hystogram showing the values of the three tests scores and the metabolism in the cluster of voxels showing statistically significant hypometabolism at PET0 as compared to PET1 (A) and viceversa (B). The mean of glucose metabolism values in the eight patients are expressed as percent of the values found in cerebellum.

Changes at PET1 in the cluster of voxel comprising mainly visual and parietal regions and significantly hypometabolic at PET0 showed an inverse trend as compared to the neuropsychological tests scores (Figure 4A). Conversely in the cluster including voxels from parahippocampal, temporal and anterior cingulate regions metabolism normalized at PET1 showing changes concordant with the neuropsychological tests scores (Figure 4B).

## Discussion

The vestibular, visual and somatosensory systems jointly perceive the spatial orientation of the human body and coordinate the processes that play a role in bringing about this orientation. It is generally accepted that this function can be ascribed to a complex network of cortical and subcortical regions that continuously process afferent inputs, with one or another element of the network possibly predominating [29].

A number of animal experiments have been performed to identify elements of such network. As a result, areas with vestibular afferentation have been found in the parietal and temporal regions of primates [2], [3].

In contrast to the accurate knowledge of the cortical representation of vestibular system resulting from electrophysiological animal studies, human data need further investigation. Functional brain activation studies over the last 20 years described the presence of separate and distinct cortical areas responsible for central processing of peripheral vestibular information [5]–[10], [16], [22], [29]–[35], and reported their activation and interactions with other sensory modalities and the changes of this network associated to strategic peripheral or central vestibular lesions [11]–[14], [18], [36].

It is already known that cortical changes induced by acute unilateral vestibular failure are various, elaborate and undergo variations over time, revealing different cortical involved areas at the onset and recovery of symptoms [11]–[14], [18], [36].

Our findings on cortical involvement during vestibular neuritis included a higher regional cerebral glucose metabolism (rCGM) in Subcallosal (BA 25), Rectal (BA 11), Inferior Frontal Gyrus (BA 47) and Posterior Insular Cortex (BA 13), as well as lower rCGM in Precuneus (BA 7), Inferior Parietal Lobule (BA 40), Somatosensory (BAs 2, 5) and Visual (BAs 17, 18, 19) cortices.

As the above-mentioned cortical areas and their role during VN have been already reported and discussed in previous studies [11]–[14], [36], we focus our discussion on the rCGM increase in two cortical regions that have never been reported in the pertinent literature: i) the Entorhinal Cortex (BAs 28, 34), when comparing PET0 to PET1 patients, and ii) the Superior Temporal Gyrus, in particular BA 38, when comparing PET0 to both PET1 and CG patients (Table 1 and 2).

### Own Body Orientation Re-encoding in the Entorhinal Cortex during Vertigo Onset in VN

Many studies showed that the Entorhinal Cortex (EC) contains a topographically organized map of the spatial environment that indicates whether the animal is positioned at one of various equally spaced locations, the orientation and distance to the environment’s walls and the location’s relative position along a common type of route. Moreover the EC works in strong connection with the hippocampus, its major output, in coding spatial navigation and spatial memory [37]–[40]. In particular it is suggested that while hippocampal *place cells* code and maintain in memory the location of specific landmarks in space, *path cells* in the EC code the direction of one’s own movements in the environment (regardless his/her actual position in space) providing the hippocampus with the needed information to compute changes in one’s own position in space [40]. Jacobs and colleagues [40] intracranially recorded EC and hippocampal cells and reported that segregated EC *path cells* discharge continuously when individuals follow a clockwise or counter clockwise direction of movement navigating in a virtual environment while hippocampal *place cells* only discharge when the individuals reach specific location along the path and when they are facing a certain direction [40].

According to theoretical models [41] the EC contributes to spatial processing and integrates geometric properties and information from the environment storing them as a durable allocentric representation in the brain. This information is used for path integration and navigation [42] in which animals or humans integrate self-movement cues (i.e., vestibular information) to locate their current position. In fact, lesions to this region determine impairment of navigation and produce a deficit in spatial memory [41], [43].

The EC receives inputs from the vestibular system and represents the major input to the hippocampus [40]. The circuitry between vestibular system, EC and hippocampus as well as its role during early phases of VN symptoms may explain the present results. It has been shown, for example, that a loss of vestibular inflow is reflected in an hippocampal atrophy [44] and that temporal inactivation of vestibular system resulted in a disruption of location-specific firing in hippocampal *place cells* [45]. The vestibular influence on hippocampal spatial representation was also demonstrated by many functional imaging studies in which vestibular stimulation [46] as well as imagined locomotion [47] and virtual navigation [48]–[51] provoked activation of right hippocampus. Moreover, Maguire et al. [48] reported an activation of right hippocampal and parahippocampal temporal regions when patients were unable to construct an internal spatial map of environment during topographical navigation.

As regards to the above-mentioned findings, we hypothesize that the found EC activation (Figure 1), only during the acute phase of VN, may be ascribed to the physiological role of this region in attempting to reorganize one’s own orientation in space, as a response to vertigo which causes an unexpected alteration of vestibular information that impairs one’s own orientation in space. Patients’ subjective scores concerning balance impairments at the early phase of vertigo onset would suggest this interpretation also. In fact, while patients reported high values of balance problems when responding to the balance questionnaire at PET0, they showed a remarkable decrease of such problems at PET1.

Thus, the EC may represent a key node in processing spatial information between the vestibular system (and other sensorimotor connected structures) and the hippocampus [44], [45]. For example, *place-cells* and *head-direction cells* in the hippocampal and parahippocampal cortices need information from the vestibular system, which may be matched in the EC where information about one’s own actual position in space (context-independent, vestibular and EC) is reorganized in terms of an external frame of reference (context-dependent, hippocampus) [37]. The physiologic behavior of EC cells is to increase their firing rate during the first minutes of exposure to a novel environment and to stabilize their firing rate thereafter in order to develop a rapid independency from the context. Hafting et al. [37] have proposed that the firing properties of the “grid cells” in the EC allow the computation of a continuously updated metric representation of the person’s location. During the early phase of vestibular neuritis, information from the vestibular system was degraded and did not allow a stabilization of these computations (and the consequent reduction of neuronal activity) as the subject would need to update its own location in space moment-by-moment resulting at the cortical level in an activation of EC that was showed at PET0. Crucially, EC cells respond differently to clockwise and counter-clockwise virtual directions of human walking paths [40] that may be considered similar to those induced by vertigo. The present findings well suite with the idea that EC neurons (path cell) encodes a perceptual, spatial or cognitive attribute of the current behavioral context. These findings support the view that the EC plays a pivotal role in memory formation because EC neurons encode the attributes of the current context that are subsequently stored by the hippocampus as memories [40].

### Early Emotional Response to Vertigo in BA 38

The BA38, located in the Superior Temporal Gyrus, was originally described by Smith, in 1907, as temporopolar cortex (TPC), then by Brodmann, in1909, as area 38, and, in 1925, by von Economo and Koskinas as area TG of von Economo and Koskinas (TG). The extent of the cortex lining this area is still and often inaccurate in functional neuroimaging studies [52].

The BA38, the most rostral portion of the human temporal lobe, has been implicated in different affective and cognitive functions such as emotion, attention, behavior regulation and memory as it is showed by the “psychic blindness” of Klüver–Bucy syndrome, due to ablation of the anterior temporal lobe; by herpetic meningoencephalitis with bilateral damage of the temporal lobes and especially of the BA 38; by neurodegenerative diseases such as Pick's disease with an important neuronal loss; in schizophrenia, especially in deficit schizophrenia and in frontal-temporal dementia [52]–[54]. The BA 38 has been studied in several experiments using PET and fMRI confirming both its involvement in different cognitive processes especially in semantic memory - Alzheimer disease declarative memory affection is associated to an atrophy of the BA 38 - in face and name recognition and in emotion processing [52], [55]–[57]. The BA 38 also seems to play an important role in coding emotional saliency after presentation of stimuli from various modalities such as olfactory, visual and auditory inputs to subjects and is thought to act within a neural network including the orbitofrontal cortex and the superior frontal gyrus (and the amygdalae) that would allow it to process information from the three sensory modalities taken together [58], [59].

As it has been showed that impairment of BA38 (i.e. due to viral damage, cortical ablation in epileptic patients, neurodegenerative disease) induces a lack of emotional responsiveness, it can be hypothesized that the activation of BA38 may be associated also to severe vertigo that would trigger an emotional response [52]–[56], [58], [59].

The activation of BA38 we report in the contrasts PET0 versus both PET1 patients (Figure 2) and CG subjects suggests that this pattern is a peculiar feature of the early phase of VN which disappears within one month as the acute symptoms fade away. Thus, we suggest that this activation is the cortical representation of the emotional component related to the vertigo symptom. This interpretation was further supported by the changes in patients’ scores in the anxiety and depersonalization/derealisation scales between vertigo onset and 1 month later. In fact, it is a common clinical observation that during the early phase of vertigo onset, patients experience some discomforts as panic, the fear of death and the absence of mental landmarks [20], [21]. Interestingly, the activation of BA38 only on the right side aligns with the emotional asymmetry theory positing that the right hemisphere may be dominant over the left one in emotional processing [60], [61].

### Correlational Analyses

Although the small sample size limits the power of a correlational approach, we run exploratory correlation analyses between the relative metabolic uptake at PET0, PET1 and the scores in the DD, Balance and Anxiety tests measured at PET0 and PET1. The correlation analyses showed an inverse relationship between the cluster resulting from the difference between PET1 and PET0 (hypometabolism during the acute phase of VN) and the scores of the DD, Balance and Anxiety scales (Fig. 4A). Conversely, a positive relationship was found between the activity of the two clusters resulting from the differences between PET0 and PET1 (hypermetabolism in the acute phase of VN) and the DD scale with a concordant trend also for Balance and Anxiety scales (Fig. 4B).

The negative correlation between DD, Balance and Anxiety and the hypometabolism at PET0 (i.e. in the acute phase of VN) suggests that a freezing in the metabolism of visual (BAs 17, 18, 19) and somatosensory (BAs 5, 7) associative cortices during disease onset was associated to a decrease of all DD, Balance and Anxiety scores. Such a metabolic response might represent an early attempt to reduce the distressing “false” primary vestibular signal [11] which may be at the neural base of the increase in DD, Balance and Anxiety scores.

Conversely, the positive correlation between the hypermetabolism at PET0 and the DD score in the cluster comprising the enthorinal (BAs 28, 34), temporal (BAs 38) and inferior-frontal cortices (BAs 11, 25 and 47) may suggest that the increase of metabolisms in these regions underpinned an increase in the perception of depersonalization symptoms during disease onset.

### Limitations

Although the results of the present study are in line with previous literature, a possible limitation of the study is the small sample size increasing the likelihood of Type II statistical errors and reducing the statistical power of the analyses. However, vestibular patients, due to the very severe symptomatology, often refused or delayed PET examination due to the discomfort to lie still in the gantry, which, together with the relative high costs of the PET/CT methodology, makes the recruitment of an inadequate number of subjects to be investigated a common limitation in functional neuroimaging studies.

We are aware that the use of a control group of subjects with a negative PET is a suboptimal solution with respect to a group of healthy volunteers. However, we would like to underline that the control group was specifically set up for this study and the same protocol and scanner were used both for patients and controls. This is of utmost importance in neuroimaging studies where the number of potential confounding variables has to be reduced to the least. The decision to use a sample of individuals with a negative PET as a control group is often due to the extremely high costs necessary to build up such cohort of subjects. Furthermore, the stringent exclusion criteria were the same ones applied in previous investigations [62], [63]. On the other hand, including as a control group neurologically normal subjects undergoing PET scan for other reasons prevents healthy individuals to be exposed to radiations and makes the technology available to most PET centers without facing the costs and the efforts necessary to build up control groups made of completely healthy subjects [64].

### Conclusions

By using FDG-PET, we showed an early involvement of EC and BA 38 in VN for the first time. We suggest that these results were not found by previous studies because our patients have been investigated in an earlier phase of symptoms onset (about two days instead of six days), when the own body spatial orientation impairments and emotional disturbances are very severe. This interpretation is further supported by the pattern of results concerning patients’ increased balance problems and anxiety levels at early stages of VN onset.

Brain functional imaging studies have not yet completely described the intricate map of cortical areas involved during vestibular failures (as those occurring in VN) and this neural network may thus be appointed as the “puzzle of vestibular cortical representation”. We believe that our findings represent a further “piece of the puzzle” and may help describing and understanding the temporal dynamics of the cortical adaptation to the symptoms associated to vestibular impairment.

## References

[1] Angelaki DE, Cullen KE (2008) Vestibular system: the many facets of a multimodal sense. Annu Rev Neurosci 31: 125–50. 18338968 10.1146/annurev.neuro.31.060407.125555

[2] Grusser OJ, Pause M, Schreiter U (1990) Localization and responses of neurons in the parieto-insular vestibular cortex of awake monkeys (Macaca fascicularis). J Physiol 430: 537–557. 2086773 10.1113/jphysiol.1990.sp018306 PMC1181752

[3] Grusser OJ, Pause M, Schreiter U (1990) Vestibular neurons in the parieto-insular cortex of monkeys (Macaca fascicularis): visual and neck receptor responses. J Physiol 430: 559–583. 2086774 10.1113/jphysiol.1990.sp018307 PMC1181753

[4] Vallar G, Perani D (1986) The anatomy of unilateral neglect after right hemisphere stroke lesions: a clinical CT correlation study in man. Neuropsychologia 24: 609–622. 3785649 10.1016/0028-3932(86)90001-1

[5] Friberg L, Olsen TS, Roland PE, Paulson OB, Lassen NA (1985) Focal increase of blood flow in the cerebral cortex of man during vestibular stimulation. Brain 108: 609–623. 3876134 10.1093/brain/108.3.609

[6] Takeda N, Hashikawa K, Moriwaki H, Oku N, Koizuka I, et al (1996) Effects of caloric vestibular stimulation on parietal and temporal blood flow in human brain: a consecutive technetium-99-HMPAO SPECT study. J Vest Res 6: 127–134. 8925115

[7] Bense S, Stephan T, Yousry TA, Brandt T, Dieterich M (2001) Multisensory cortical signal increases and decreases during vestibular galvanic stimulation (fMRI). J Neurophysiol 85: 886–899. 11160520 10.1152/jn.2001.85.2.886

[8] Suzuki M, Kitano H, Ito R, Kitanishi T, Yazawa Y, et al (2001) Cortical and subcortical vestibular response to caloric stimulation detected by functional magnetic resonance imaging. Brain Res Cogn Brain Res 12: 441–449. 11689304 10.1016/s0926-6410(01)00080-5

[9] Wenzel R, Bartenstein P, Dieterich M, Danek A, Weindl A, et al (1996) Deactivation of human visual cortex during involuntary ocular oscillations. A PET activation study. Brain 119: 101–110. 8624674 10.1093/brain/119.1.101

[10] Bottini G, Karnath HO, Vallar G, Sterzi R, Frith CD, et al (2001) Cerebral representations for egocentric space. Functional anatomical evidence from caloric vestibular stimulation and neck vibration. Brain 124: 1182–1196. 11353734 10.1093/brain/124.6.1182

[11] Bense S, Bartenstein P, Lochmann M, Schlindwein P, Brandt T, et al (2004) Metabolic changes in vestibular and visual cortices in acute vestibular neuritis. Ann Neurol 56: 624–630. 15449325 10.1002/ana.20244

[12] Dieterich M, Brandt T (2008) Functional brain imaging of peripheral and central vestibular disorders. Brain 131(10): 2538–52. 18515323 10.1093/brain/awn042

[13] Dieterich M, Brandt T (2010) Imaging cortical activity after vestibular lesions. Restor Neurol Neurosci 28(1): 47–56. 20086282 10.3233/RNN-2010-0505

[14] Helmchen C, Klinkenstein J, Machner B, Rambold H, Mohr C, et al (2009) Structural changes in the human brain following vestibular neuritis indicate central vestibular compensation. Ann N Y Acad Sci 1164: 104–15. 19645887 10.1111/j.1749-6632.2008.03745.x

[15] Lopez C, Blanke O (2011) The thalamocortical vestibular system in animals and humans. Brain Res Rev 67(1–2): 119–46. 21223979 10.1016/j.brainresrev.2010.12.002

[16] zu Eulenburg P, Caspers S, Roski C, Eickhoff SB (2012) Meta-analytical definition and functional connectivity of the human vestibular cortex. Neuroimage 23 60(1): 162–169. 10.1016/j.neuroimage.2011.12.032 22209784

[17] Alessandrini M, D’Erme G, Bruno E, Napolitano B, Magrini A (2003) Vestibular compensation: analysis of postural rearrangement as a control index for unilateral vestibular deficit. Neuroreport 14: 1–5. 12802206 10.1097/01.wnr.0000070827.57864.49

[18] Alessandrini M, Napolitano B, Bruno E, Belcastro L, Ottaviani F, et al (2009) Cerebral plasticity in acute vestibular deficit. Eur Arch Otorhinolaryngol 266(10): 1547–51. 19294399 10.1007/s00405-009-0953-4

[19] Strupp M, Brandt T (2009) Vestibular neuritis. Semin Neurol 29(5): 509–19. 19834862 10.1055/s-0029-1241040

[20] Pollak L, Klein C, Rafael S, Vera K, Rabey JM (2003) Anxiety in the first attack of vertigo. Otolaryngol Head Neck Surg 128(6): 829–34. 12825034 10.1016/S0194-59980300454-6

[21] Tschan R, Wiltink J, Best C, Bense S, Dieterich M, et al (2008) Validation of the German version of the Vertigo Symptom Scale (VSS) in patients with organic or somatoform dizziness and healthy controls. J Neurol 255(8): 1168–75. 18481033 10.1007/s00415-008-0863-1

[22] zu Eulenburg P, Stoeter P, Dieterich M (2010) Voxel-based morphometry depicts central compensation after vestibular neuritis. Ann Neurol 68(2): 241–9. 20695016 10.1002/ana.22063

[23] Gomez-Alvarez FB, Jauregui-Renaud K (2011) Psychological symptoms and spatial orientation during the first 3 months after acute unilateral vestibular lesion. Arch Med Res 42(2): 97–103. 21565621 10.1016/j.arcmed.2011.03.004

[24] Zung WK (1971) A rating instrument for anxiety disorders. Psychosomatics 12: 371–379. 5172928 10.1016/S0033-3182(71)71479-0

[25] Cox BJ, Swinson RP (2002) Instrument to assess depersonalization/derealization in panic disorder. Depress Anxiety 15: 172–175. 12112722 10.1002/da.10051

[26] Cooper CW (1993) Vestibular neuronitis: a review of a common cause of vertigo in general practice. Br J Gen Pract 43(369): 164–7. 8323804 PMC1372362

[27] Cistaro A, Valentini MC, Chiò A, Nobili F, Calvo A, et al (2011) Positron Emission Tomography discriminates bulbar and spinal onset in Amyotrophic Lateral Sclerosis. Eur J Nuc Med Mol Imaging 39: 251–259. 10.1007/s00259-011-1979-6 22089661

[28] Varrone A, Asenbaum S, Vander Borght T, Booij J, Nobili F, et al (2009) EANM procedure guidelines for PET brain imaging using [18F]FDG, version 2. Eur J Nucl Med Mol Imaging 36(12): 2103–10. 19838705 10.1007/s00259-009-1264-0

[29] Guldin WO, Grusser OJ (1998) Is there a vestibular cortex? Trends Neurosci 21: 254–259. 9641538 10.1016/s0166-2236(97)01211-3

[30] Vallar G, Bottini G, Rusconi ML, Sterzi R (1993) Exploring somatosensory hemineglect by vestibular stimulation. Brain 116: 71–86. 8453466 10.1093/brain/116.1.71

[31] Fasold O, von Brevern M, Kuhberg M, Ploner CJ, Villringer A, et al (2002) Human vestibular cortex as identified with caloric stimulation in functional magnetic resonance imaging. Neuroimage 17: 1384–1393. 12414278 10.1006/nimg.2002.1241

[32] Eickhoff SB, Weiss PH, Amunts K, Fink GR, Zilles K (2006) Identifying human parieto-insular vestibular cortex using fMRI and cytoarchitectonic mapping. Hum Brain Mapp 27(7): 611–21. 16281284 10.1002/hbm.20205 PMC6871353

[33] Dieterich M, Bartenstein P, Spiegel S, Bense S, Schwaiger M, et al (2005) Thalamic infarctions cause side-specific suppression of vestibular cortex activations. Brain 128: 2052–2067. 15947061 10.1093/brain/awh551

[34] Stephan T, Deutschländer A, Nolte A, Schneider E, Wiesmann M, et al (2005) Functional MRI of galvanic vestibular stimulation with alternating currents at different frequencies. Neuroimage 26: 721–732. 15955481 10.1016/j.neuroimage.2005.02.049

[35] Schlindwein P, Mueller M, Bauermann T, Stoeter P, Dieterich M (2008) Cortical representation of saccular vestibular stimulation: VEMPs in fMRI. Neuroimage 39: 19–31. 17919936 10.1016/j.neuroimage.2007.08.016

[36] Brandt T, Bartenstein P, Janek A, Dieterich M (1998) Reciprocal inhibitory visual-vestibular interaction. Visual motion stimulation deactivates the parieto-insular vestibular cortex. Brain 121: 1749–1758. 9762962 10.1093/brain/121.9.1749

[37] Hafting T, Fyhn M, Molden S, Moser M, Moser E (2005) Microstructure of a spatial map in the entorhinal cortex. Nature 436(7052): 801–6. 15965463 10.1038/nature03721

[38] Hargreaves E, Rao G, Lee I, Knierim J (2005) Major dissociation between medial and lateral entorhinal input to dorsal hippocampus. Science 308(5729): 1792–4. 15961670 10.1126/science.1110449

[39] Loureiro M, Lecourtier L, Engeln M, Lopez J, Cosquer B, et al (2012) The ventral hippocampus is necessary for expressing a spatial memory. Brain Struct Funct 217(1): 93–106. 21667304 10.1007/s00429-011-0332-y

[40] Jacobs J, Kahana MJ, Ekstrom AD, Mollison MV, Fried I (2010) A sense of direction in human entorhinal cortex”. Proc Natl Acad Sci U S A 107(14): 6487–6492. 20308554 10.1073/pnas.0911213107 PMC2851993

[41] Parron C, Poucet B, Save E (2004) Entorhinal cortex lesions impair the use of distal but not proximal landmarks during place navigation in the rat. Behavioural Brain Research 154: 345–352. 15313022 10.1016/j.bbr.2004.03.006

[42] Fyhn M, Molden S, Witter MP, Moser EI, Moser M (2004) Spatial representation in the entorhinal cortex. Science 305: 1258–1264. 15333832 10.1126/science.1099901

[43] Nagahara HA, Otto T, Gallagher M (1995) Entorhinal-perirhinal lesions impair performance of rats on two versions of place learning in the Morris water maze. Behavioral Neuroscience 109(1): 3–9. 7734077 10.1037//0735-7044.109.1.3

[44] Hartley T, Maguire EA, Spiers HJ, Burgess N (2003) The well-worn route and the path less traveled: distinct neural bases of route following and wayfinding in humans. Neuron 37: 877–88. 12628177 10.1016/s0896-6273(03)00095-3

[45] Maguire EA, Valentine ER, Wilding JM, Kapur N (2003) Routes to remembering: the brains behind superior memory. Nat Neurosci 6: 90–5. 12483214 10.1038/nn988

[46] Vitte E, Derosier C, Caritu Y, Berthoz A, Hasboun D, et al (1996) Activation of the hippocampal formation by vestibular stimulation: a functional magnetic resonance imaging study. Exp Brain Res 112: 523–6. 9007554 10.1007/BF00227958

[47] Jahn K, Deutschlander A, Stephan T, Strupp M, Wiesmann M, et al (2004) Brain activation patterns during imagined stance and locomotion in functional magnetic resonance imaging. Neuroimage 22: 1722–31. 15275928 10.1016/j.neuroimage.2004.05.017

[48] Maguire EA, Burke T, Phillips J, Staunton H (1996) Topographical disorientation following unilateral temporal lobe lesions in humans. Neuropsychologia 34: 993–1001. 8843066 10.1016/0028-3932(96)00022-x

[49] Maguire EA, Frackowiak RS, Frith CD (1997) Recalling routes around London: activation of the right hippocampus in taxi drivers. J Neurosci 17: 7103–10. 9278544 10.1523/JNEUROSCI.17-18-07103.1997 PMC6573257

[50] Gron G, Wunderlich AP, Spitzer M, Tomczak R, Riepe MW (2000) Brain activation during human navigation: gender-different neural networks as substrate of performance. Nat Neurosci 3: 404–8. 10725932 10.1038/73980

[51] Brandt T, Schautzer F, Hamilton DA, Brüning R, Markowitsch HJ, et al (2005) Vestibular loss causes hippocampal atrophy and impaired spatial memory in humans. Brain 128(11): 2732–41. 16141283 10.1093/brain/awh617

[52] Blaizot X, Mansilla F, Insausti AM, Constans JM, Salinas-Alamán A, et al (2010) The human parahippocampal region: I. Temporal pole cytoarchitectonic and MRI correlation. Cereb Cortex 20(9): 2198–212. 20064939 10.1093/cercor/bhp289 PMC2923216

[53] Dupont S (2002) Investigating temporal pole function by functional imaging. Epileptic Disord 4: S17–S22. 12424086

[54] Olson IR, Plotzker A, Ezzyat Y (2007) The Enigmatic temporal pole: a review of findings on social and emotional processing. Brain 130: 1718–1731. 17392317 10.1093/brain/awm052

[55] Corkin S, Amaral DG, Gonzalez RG, Johnson KA, Hyman BT (1997) H.M.’s medial temporal lobe lesion: findings from magnetic resonance imaging. J Neurosci 17: 3964–3979. 9133414 10.1523/JNEUROSCI.17-10-03964.1997 PMC6573687

[56] Dolan RJ, Lane R, Chua P, Fletcher P (2000) Dissociable temporal lobe activations during emotional episodic memory retrieval. NeuroImage 11: 203–209. 10694462 10.1006/nimg.2000.0538

[57] Rankin KP, GornoTempini ML, Allison SC, Stanley CM, Glenn S, et al (2006) Structural anatomy of empathy in neurodegenerative disease. Brain 129: 2945–2956. 17008334 10.1093/brain/awl254 PMC2562652

[58] Royet JP, Zald D, Versace R, Costes N, Lavenne F, et al (2000) Emotional responses to pleasant and unpleasant olfactory, visual, and auditory stimuli: a positron emission tomography study. J Neurosci 20: 7752–7759. 11027238 10.1523/JNEUROSCI.20-20-07752.2000 PMC6772882

[59] Zald DH, Pardo JV (2002) The neural correlates of aversive auditory stimulation. NeuroImage 16: 746–753. 12169258 10.1006/nimg.2002.1115

[60] Davidson RJ (1998) Affective style and affective disorders: perspectives from affective neuroscience. Cogn Emot 12: 307–330.

[61] Cope LM, Schaich Borg J, Harenski CL, Sinnott-Armstrong W, Lieberman D, et al (2010) Hemispheric asymmetries during processing of immoral stimuli. Front Evol Neurosci 2: 110. 21344009 10.3389/fnevo.2010.00110 PMC3034229

[62] Pagani M, Dessi B, Morbelli S, Brugnolo A, Salmaso D, et al (2010) MCI patients declining and not-declining at mid-term follow-up: FDG-PET findings. Curr Alzheimer Res. 7: 287–294. 10.2174/156720510791162368 19939228

[63] Nobili F, Mazzei D, Dessi B, Morbelli S, Brugnolo A, et al (2010) Unawareness of memory deficit in amnestic MCI: FDG-PET findings. J Alzheimers Dis. 22: 993–1003. 10.3233/JAD-2010-100423 20858977

[64] Del Sole A, Clerici F, Chiti A, Lecchi M, Mariani C, et al (2008) Individual cerebral metabolic deficits in Alzheimer’s disease and amnestic mild cognitive impairment: an FDG PET study. Eur J Nucl Med Mol Imaging. 35: 1357–13. 10.1007/s00259-008-0773-6 18418593

